# Effects of Peroxisome Proliferator-Activated Receptor-δ Agonist on Cardiac Healing after Myocardial Infarction

**DOI:** 10.1371/journal.pone.0148510

**Published:** 2016-02-10

**Authors:** Jeong Rang Park, Jong Hwa Ahn, Myeong Hee Jung, Jin-Sin Koh, Yongwhi Park, Seok-Jae Hwang, Young-Hoon Jeong, Choong Hwan Kwak, Young Soo Lee, Han Geuk Seo, Jin Hyun Kim, Jin-Yong Hwang

**Affiliations:** 1 Division of Cardiology, Department of Internal Medicine, Gyeongsang National University School of Medicine and Gyeongsang National University Hospital, Jinju, Republic of Korea; 2 Institute of Health Sciences, Gyeongsang National University, Jinju, Republic of Korea; 3 Biomedical Research Institute, Gyeongsang National University Hospital, Jinju, Republic of Korea; 4 Department of Pharmacology, Gyeongsang National University School of Medicine, Jinju, Republic of Korea; 5 Department of Animal Biotechnology, Konkuk University, Seoul, Republic of Korea; Nihon University School of Medicine, JAPAN

## Abstract

Peroxisome proliferator-activated receptor-delta (PPAR-δ)-dependent signaling is associated with rapid wound healing in the skin. Here, we investigated the therapeutic effects of PPAR-δ-agonist treatment on cardiac healing in post-myocardial infarction (MI) rats. Animals were assigned to the following groups: sham-operated control group, left anterior descending coronary artery ligation (MI) group, or MI with administration of the PPAR-δ agonist GW610742 group. GW610742 (1 mg/kg) was administrated intraperitoneally after the operation and repeated every 3 days. Echocardiographic data showed no differences between the two groups in terms of cardiac function and remodeling until 4 weeks. However, the degrees of angiogenesis and fibrosis after MI were significantly higher in the GW610742-treated rats than in the untreated MI rats at 1 week following MI, which changes were not different at 2 weeks after MI. Naturally, PPAR-δ expression in infarcted myocardium was highest increased in 3 day after MI and then disappeared in 14 day after MI. GW610742 increased myofibroblast differentiation and transforming growth factor-beta 2 expression in the infarct zone at 7 days after MI. GW610742 also increased bone marrow-derived mesenchymal stem cell (MSC) recruitment in whole myocardium, and increased serum platelet-derived growth factor B, stromal-derived factor-1 alpha, and matrix metallopeptidase 9 levels at day 3 after MI. PPAR-δ agonists treatment have the temporal effect on early fibrosis of infarcted myocardium, which might not sustain the functional and structural beneficial effect.

## Introduction

The mortality rate of patients with acute myocardial infarction (MI) has dramatically improved due to the development of timely revascularization treatments. However, MI is still the most frequent cause of heart failure worldwide. The healing process after MI involves repairing the infarcted myocardium, and is intertwined with left ventricular (LV) remodeling, which can result in heart failure [1]. During this healing process, the infarcted myocardium undergoes a series of cellular, molecular, histological, and extracellular responses, and can be divided into three overlapping phases: inflammation, proliferation, and maturation, which occur in a timed sequence [1,2]. The inflammation phase, the first step after MI, is characterized by degradation of the extracellular matrix, inhibition of tissue proliferation, and release of inflammatory mediators [3]. During this period, the infarcted myocardium is vulnerable to the mechanical stress of cyclic intraventricular pressure and myocardial contractility. To compensate for the weakened myocardial architecture, collagen is deposited as fibrosis begins [4,5]. Fibrosis can attenuate inflammation and dilatation of the infarcted heart. A prolonged inflammatory phase and deficient fibrosis can lead to free wall rupture, aneurysmal dilatation, and late heart failure [6,7]. Acceleration of the healing process, particularly fibrosis, is thus clinically critical for preventing cardiac rupture and progressive remodeling after MI.

Peroxisome proliferator-activated receptors (PPARs) constitute a subfamily of ligand-activated transcription factors that belong to the nuclear hormone receptor superfamily. There are three members of the PPAR subfamily: PPAR-α, PPAR-β/δ (PPAR-δ), and PPAR-γ [8]. The PPAR-δ mRNA level is higher than the mRNA levels of PPAR-α and PPAR-γ in rat neonatal and adult cardiac fibroblasts, and that PPAR-δ is functionally the most important isoform in cardiac fibroblasts and cardiac myofibroblasts [8, 9]. The anti-apoptotic and anti-inflammatory effects of PPAR-δ agonists have been demonstrated in rat models of myocardial ischemia/reperfusion injury [10,11]. PPAR-δ is also known to play a crucial role in physiological angiogenesis in a mouse hindlimb ischemia, in a mouse skin punch wound, and a mouse corneal injury model [12, 13]. We hypothesized that PPAR-δ agonist treatment may have beneficial effect on cardiac healing after MI. However, the role of PPAR-δ in the *in vivo* cardiac healing process has not been investigated.

Therefore, this study was aimed to examine the potential of PPAR-δ agonist as a therapeutic drug for MI healing and to investigate the effects of the PPAR-δ agonist GW610742 on early healing in MI model with a left anterior descending coronary artery (LAD) ligation in rats.

## Materials and Methods

### Ethics Statement

The experiments were approved (GLA-110324-R0022) by the Gyeongsang National University Institution Animal Care & Use Committee.

### Animals and Surgery

This study was conducted using male Sprague-Dawley (SD) rats (230–250 g; Koatech Inc., Peongtaek, Korea). Animals were housed in temperature-controlled conditions under a light/dark photocycle with food and water supplied *ad libitum*. The experiments were performed according to the Gyeongsang National University Animal Care and Use Committee guidelines (GLA-110324-R0022). MI was induced in anesthetized rats via ligation of the LAD. The rats were scarified after heart harvest under anesthesia. Anesthesia for surgery and heart harvest was induced by intraperitoneal injection of mixture of zoletil (Virbac Korea, Seoul, Korea) and rompun (Bayer Korea, Seoul, Korea).

The PPAR-δ agonist GW610742 (GlaxoSmithKline, Stevenage, UK) was immediately injected intraperitoneally. The doses of GW610742 (1 mg/kg) were based on previous studies [10,14], and the injections were repeated every 3 days. The rats were divided into three groups: (1) thoracotomy without LAD ligation (sham-operated group, n = 20); (2) thoracotomy with LAD ligation (MI group, n = 35); and (3) thoracotomy with LAD ligation and treatment with GW610742 (MI + GW group, n = 35). Based on echocardiographic images, hearts with the same degree of infarction were used for all experimental procedures.

### Echocardiography

Transthoracic echocardiography was performed at post-operation and sacrifice. Two-dimensional and M-mode echocardiograms were assessed using a 12-MHz linear array transducer with a VIVID Q system (GE Healthcare, Wauwatosa, WI, USA). M-mode images for the LV fractional shortening, LV ejection fraction, stroke volume (SV), LV dimension at end-diastole, LV dimension at end-systole, diastolic interventricular septal thickness (IVS), and LV mass were acquired at the level of the papillary muscle of the LV. The infarction size was calculated by the percentage of akinetic portion to total LV perimeter at single short axis image of papillary muscle level [15].

### Verification of tissue fibrosis

To analyze the collagen deposition, the sections were stained with Masson’s trichrome stain. Ten infarcted zones, randomly selected at 400× magnification, were assessed in each rat, and the density of the trichrome-positive signals was analyzed using NIS-Elements BR 3.2 (Nikon, Japan).

### Protein and mRNA expression

Heart tissues were suspended in a protein lysis buffer; then, the proteins were transferred to nitrocellulose membranes. Blots were probed using the following primary antibodies: polyclonal anti-transforming growth factor-beta 2 (TGF-β2), anti-collagen I (Calbiochem, Darmstadt, Germany), anti-PPAR-δ (Abcam, Cambridge, UK), anti-osteoblast cadherin (OB-cad) (Cell Signaling, Dallas, TX, USA), anti-α-smooth muscle actin (anti-α-SMA; Sigma-Aldrich, Saint Louis, MO, USA), anti-Ki67 (Abcam, Cambridge, UK), and anti-CD31 (AbD Serotec, Raleigh, NC, USA). The blots were visualized using secondary antibodies with an enhanced chemiluminescence kit. Northern blot analysis was employed for investigating TGF-β1 and -β2 mRNA expression.

### Immunohistochemical analysis

Immunohistochemistry using the Vectastain ABC kit (Vector Laboratories, CA, USA) was performed on 5-μm-thick sections of paraffin-embedded heart tissue. Sections were blocked with 1% normal goat serum; treated successively with anti-collagen I, -CD31, and -Ki-67 antibodies at 4°C overnight; incubated for 90 min at room temperature with secondary antibody; and finally, incubated with the avidin-biotinylated-horseradish-peroxidase-complex for 60 min at room temperature, rinsed in phosphate buffered saline, and developed using 3,3'-diaminobenzidine tetrahydrochloride.

### Analysis of mesenchymal stem cell (MSC) phenotype in the infarcted heart

Rat cardiac MSCs were prepared from the ventricles of six adult male SD rats. Procedures were slightly modified from those in previous descriptions [16,17]. The ventricles were pooled, minced, and digested with type II collagenase solution. Cells were incubated in a culture dish at 37°C for 1 h; floating cells were removed and adherent cells were used for MSC phenotype analysis. Adherent cells were stained with each antibody (CD29, CD44, CD45, CD90, and CD105; Miltenyi Biotec Co., Bergisch Gladbach, Germany) and positive cells were verified by flow cytometry.

### Measurement of serum platelet-derived growth factor B (PDGF-b), matrix metallopeptidase 9 (MMP-9), basic fibroblast growth factor (bFGF), stromal-derived factor-1 alpha (SDF-1α), and vascular endothelial growth factor (VEGF)

Serum levels of PDGF-b, MMP-9, bFGF, SDF-1α and VEGF were quantified using specific enzyme-linked immunosorbent assay kits (ELISA) according to the manufacturer’s instructions (Biosource International, Nivelles, Belgium).

### Statistical analysis

Statistical analysis was performed using the SPSS software (SPSS Interactive Graphics, Version 19.0, SPSS Inc, Chicago, IL, USA), and *P* < 0.05 was considered statistically significant. Statistical differences between the experimental groups were determined using analyses of variance and Student’s *t*-tests. Values are represented as the mean ± the standard error of the mean.

## Results

### Structural and functional LV changes

**Fig 1:**
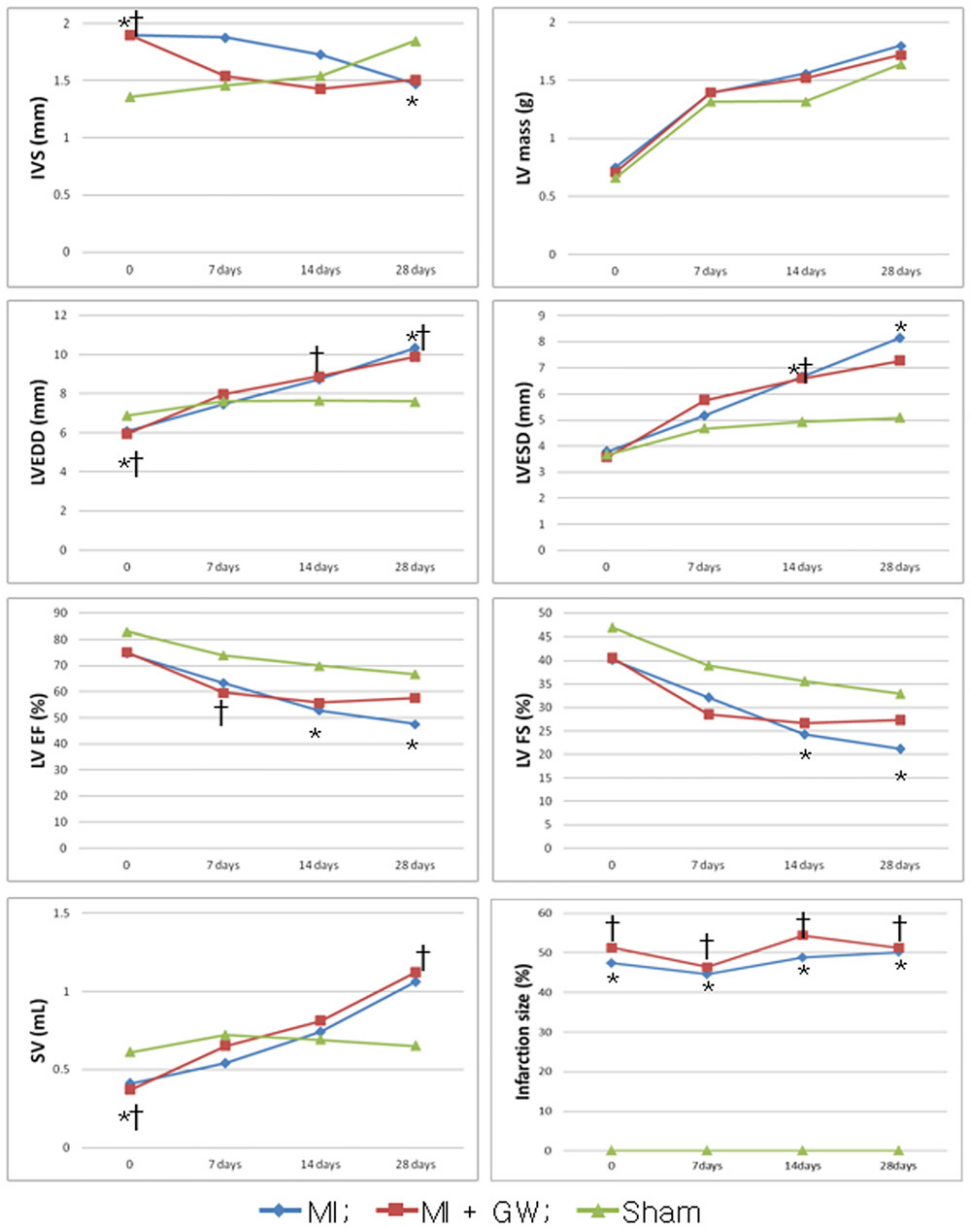
Echocardiographic measurements of left ventricular remodeling. The consecutive measurements of the echocardiographic parameters were acquired at baseline (0, immediate post-operation), 7 days, 14 days, and 28 days after surgical procedure. *P <0.05 between the MI group vs. sham group. †*P* <0.05 between the MI + GW group vs. sham group. MI, myocardial infarction only; MI + GW, MI treated with GW610742, IVS, interventricular septal thickness; LVEDD, left ventricular dimension at end-diastole; LVESD, left ventricular dimension at end-systole; FS, fractional shortening; EF, ejection fraction; SV, stroke volume. Sham (n = 3/each day), MI (n = 28 of baseline, n = 18 of 7days, n = 17 of 14 days, n = 6 of 28days), MI + GW (n = 31 of baseline, n = 19 of 7days, n = 15 of 14 days, n = 6 of 28days).

Echocardiographic assessments were performed to evaluate the geometry and functional changes in the LV (Fig 1). These measures were followed serially during the 28-day experimental period, and we compared the values between the MI + GW and MI groups. The infarction size by percent of LV perimeter using echocardiography (Fig 1) was not significant change with time and not different between the MI + GW and MI groups. The infarct area, calculated by % fibrotic area of the LV on Masson’s trichrome stain, were not different between both groups (S1 Fig). Immediate post-procedure IVS of both MI + GW and MI groups were thicker than that of sham (1.90 ± 0.37 mm and 1.90 ± 0.49 mm vs. 1.36 ± 0.13 mm, p <0.001) because of injury related inflammation and edema. After 7 days, MI + GW group was showed a tendency to rapidly improve IVS than MI group (1.54 ± 0.39 mm and 1.88 ± 0.70 mm, p = 0.065). However, none of the parameters was significantly different between the MI+GW and MI groups (Fig 1).

### The Effect of PPAR-δ agonist GW610742 on fibrosis and angiogenesis in the infarcted hearts

**Fig 2:**
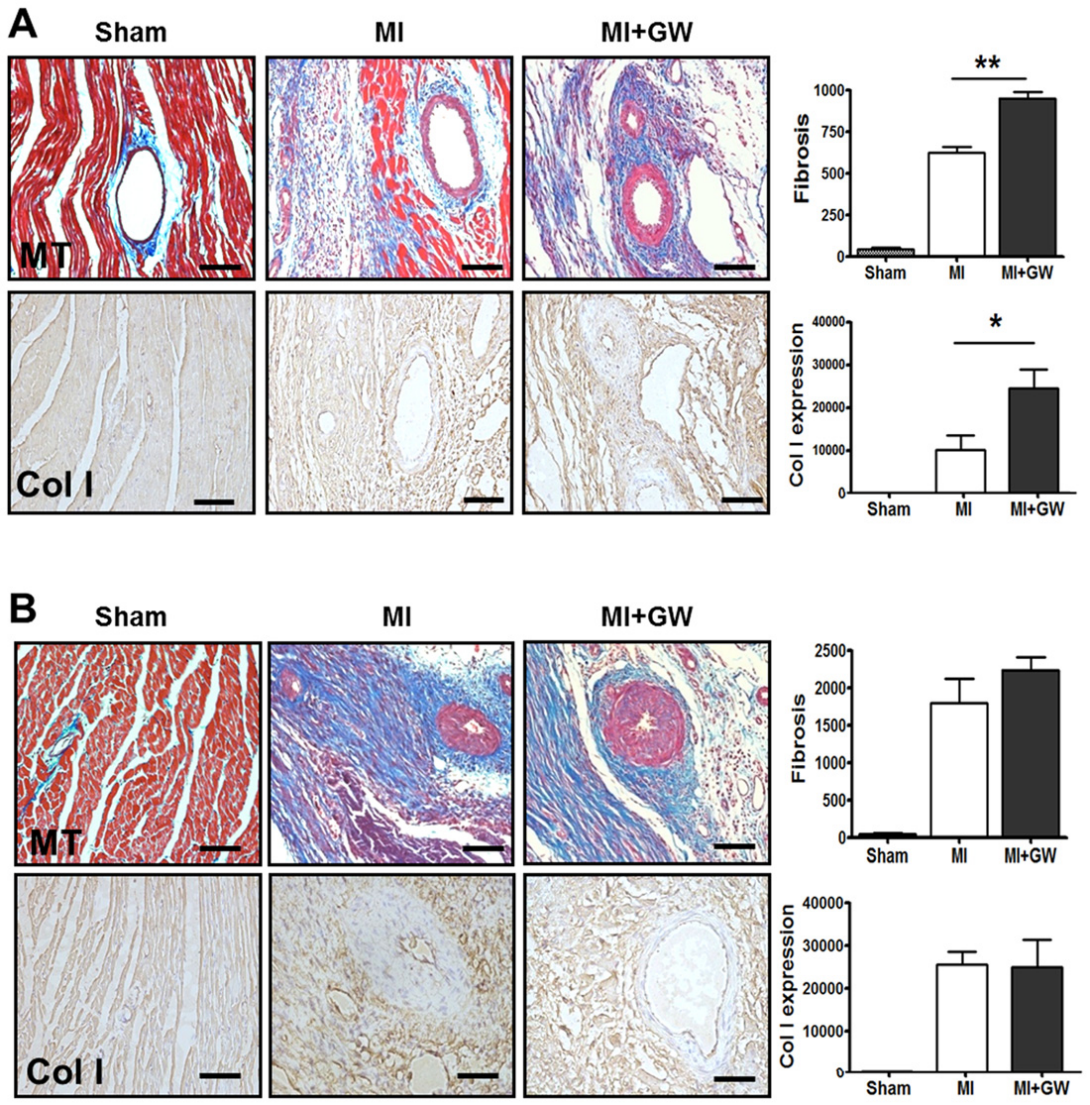
Effects of the PPAR-δ agonist GW610742 on fibrosis. Representative images from sham-operated (Sham), myocardial infarction alone (MI), and MI treated with GW610742 (MI + GW) rats on day 7 **(A)** and day 14 **(B)** after MI. Densitometric analysis shows MT staining, and collagen I-stained tissue, confirming the differences in fibrosis between the groups. Values are represented as the mean ± the standard error of the mean. *P < 0.05 and **P < 0.01 vs. the corresponding MI group. Scale bars = 100 μm. Sham, sham-operated; MI, myocardial infarction only; MI+GW, MI treated with GW610742; MT, Masson’s trichrome stain; Col I, collagen-1. Sham (n = 3/each day), MI (n = 7/each day), MI + GW (n = 7/each day).

A critical event during cardiac healing is extensive fibrosis. To determine the role of PPAR-δ in the infarcted heart tissue, we investigated the histological changes after MI. Prominent fibrosis began to be found on day 0 and day 3 in both MI and MI + GW groups but not significant between the MI and MI + GW groups (S2 Fig). As shown in Fig 2, the degree of fibrosis and number of collagen I-positive cells were greater in the MI + GW group compared to in the MI group at day 7 (Fig 2A). The degree of fibrosis and number of collagen I-positive cells were also detected at day 14 and were stronger than those detected at day 7, but no significant differences were identified between the MI only and MI + GW groups (Fig 2B).

**Fig 3:**
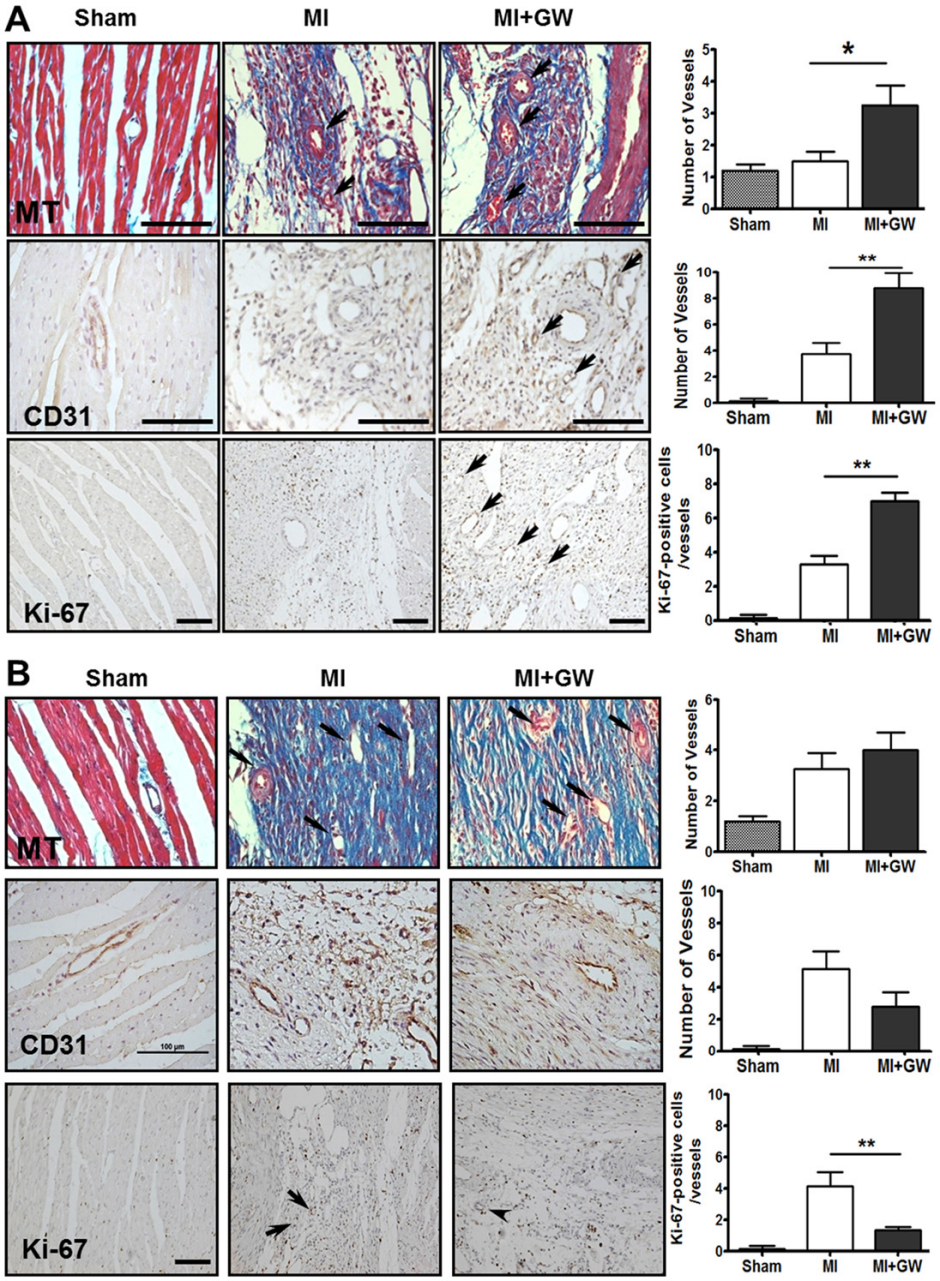
Effects of the PPAR-δ agonist GW610742 on angiogenesis. Representative images from the sham-operated (Sham), myocardial infarction alone (MI), and MI treated with GW610742 (MI + GW) groups on day 7 **(A)** and day 14 **(B)** post-surgery. Images using Masson’s trichrome staining (MT) show vessels (arrows) in the heart post-operation. The immunoreactivity and positive cell numbers for CD31 and Ki-67 (arrows) were counted in each group post-operation. Values are represented as the mean ± the standard error of the mean (SEM). *P < 0.05 and vs. **P < 0.01 the corresponding MI group. Scale bars = 100 μm and 50 μm for MT, CD31, and Ki-67 staining, respectively. Sham (n = 3/each day), MI (n = 7/each day), MI + GW (n = 7/each day).

We also analyzed the differences in angiogenesis, another critical process that occurs during healing and repair, in the infarcted heart tissues. Sections from the MI + GW group had significantly more vessels than did sections from the MI group at 7 days after MI (Fig 3A). To confirm whether PPAR-δ is associated with angiogenesis during LV healing, sections were stained with anti-CD31. CD31 immunoreactivity was observed in the endothelium of regenerating and mature vessels and was significantly more abundant in the MI + GW group than in the MI group at day 7 after MI (Fig 3A). Because angiogenesis is accompanied by endothelial cell (EC) proliferation, sections were stained with anti-Ki-67 antibodies. Most of the Ki-67 immunoreactivity was observed in the endothelium of regenerating vessels in the infarcted hearts from the MI and MI + GW groups. The Ki-67 signals were more dense and higher in the infarcted hearts from the MI + GW group at day 7 (Fig 3A). Interestingly, the Ki-67 positive cells were decreased in MI + GW group compared with MI alone group at day 14 (Fig 3B). On day 0 and day 3, no significant changes were found between the MI and MI + GW groups (S3 Fig).

### Expression of PPAR-δ in the infarcted hearts

**Fig 4:**
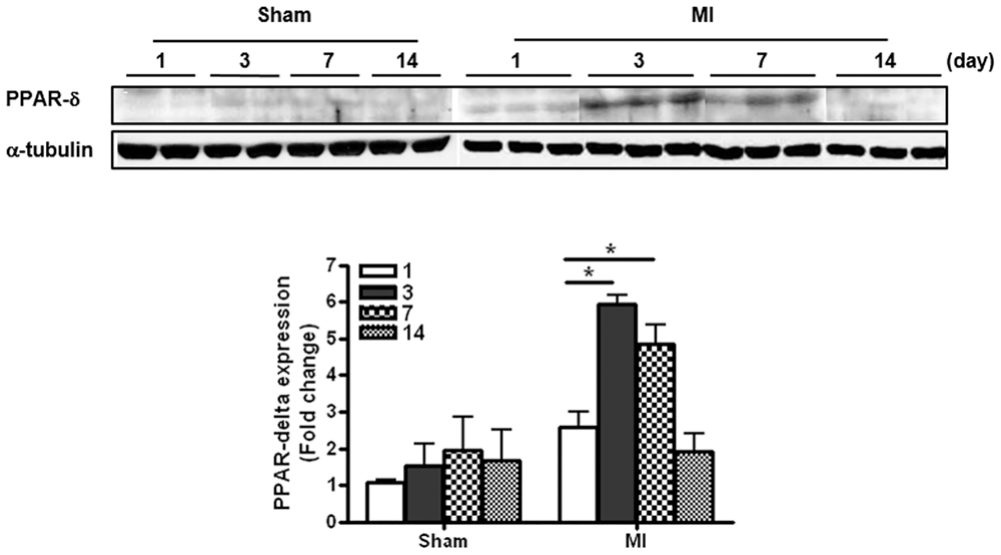
Expression of PPAR-δ in the infarcted zone. PPAR-δ protein expression in the infarct zone in a heart with MI. Representative blots for PPAR-δ expression from sham-operated (Sham) rats and rats with MI alone (MI) on day 1 through day 14 post-surgery. *P < 0.05. PPAR-δ, peroxisome proliferator-activated receptor-delta; Sham, sham-operated; MI, myocardial infarction. Sham (n = 4/each day), MI (n = 10/each day).

To investigate of mechanism of PPAR- δ agonist on the infarcted heart, we tried to know the natural expression of PPAR-δ in MI only model. As the results, PPAR-δ expression was increased in the infarcted heart, and the level peaked at day 3 and then gradually decreased on days 7 and 14 (Fig 4). However, the level was unchanged in the non-infarcted heart tissue (data not shown) and the increased PPAR-δ expression was variable in the infarcted heart treated with GW610742 (S4 Fig).

### GW610742 increases the expression of TGF-β2

**Fig 5:**
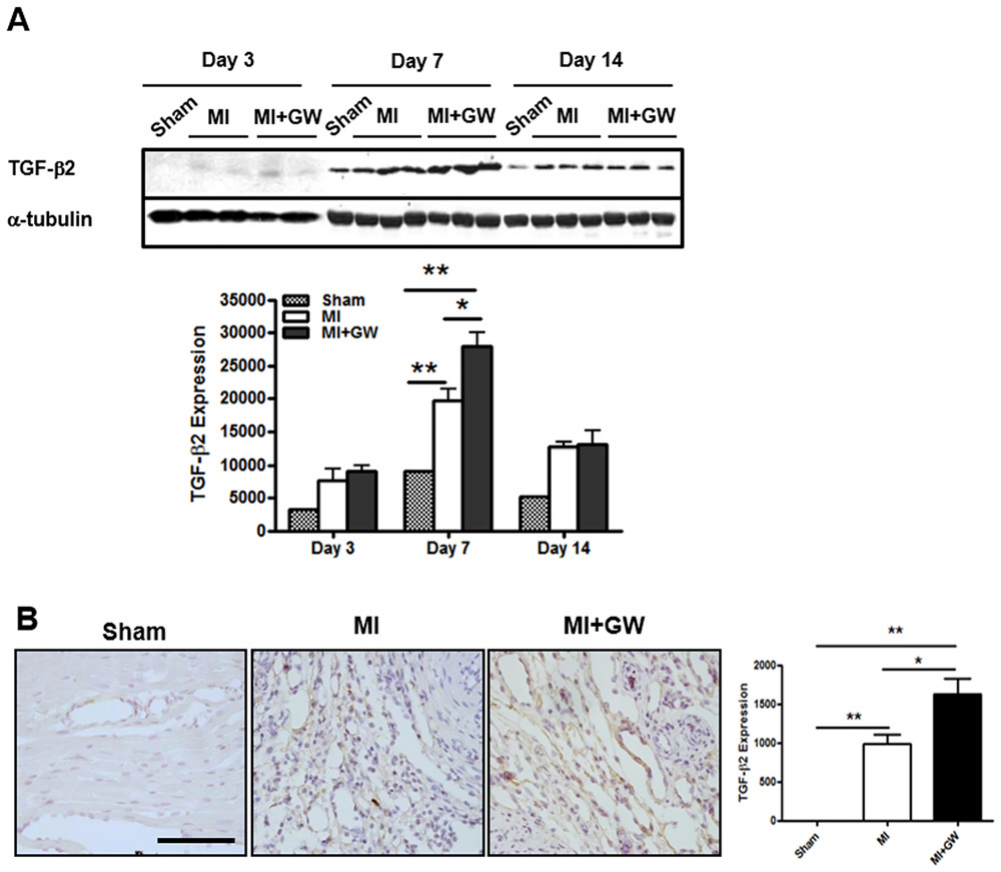
Effects of GW610742 on TGF-β2 expression. **(A)** TGF-β2 (12.5 kDa) expression was assessed by immunoblot analyses in all groups on day 3, 7, and day 14 following surgery. Densitometric analysis shows the relative levels of TGF-β2 expression in each group. α-tubulin (42 kDa), used as a loading control, was not different between the groups. Representative blots are derived from three separate experiments. Values are represented as the mean ± SEM. *P < 0.05 and **P < 0.01. Sham (n = 3), MI (n = 10), MI + GW (n = 10). (**B)** Representative immunohistochemical images for TGF-β2 from the MI alone (MI) group and the MI treated with GW610742 (MI + GW) group on day 7 post-surgery. Scale bars = 100 μm. TGF-β2, transforming growth factor-beta 2.

Several reports have suggested that TGF-β is a target molecule of PPAR-δ [18,19]. Moreover, this molecule is known as a mediator of fibrosis and angiogenesis in LV healing after MI [20]. Therefore, we examined the expression change of TGF-β after PPAR-δ activation in the infarct site. Relative to the expression in controls, TGF-β2 expression was significantly increased following MI in the infarcted hearts at day 7 after MI. The levels were significantly greater in the MI + GW group than they were in the MI group (Fig 5A and 5B). In contrast to TGF-β2 expression, the levels of TGF-β1 were not significantly different between the MI + GW and MI groups (S5 Fig). These results suggest that TGF-β2 expression, compared to TGF-β1 expression, was more sensitive to the PPAR-δ agonist in fibrosis.

### GW610742 increases the population of MSCs in the infarcted hearts

**Fig 6:**
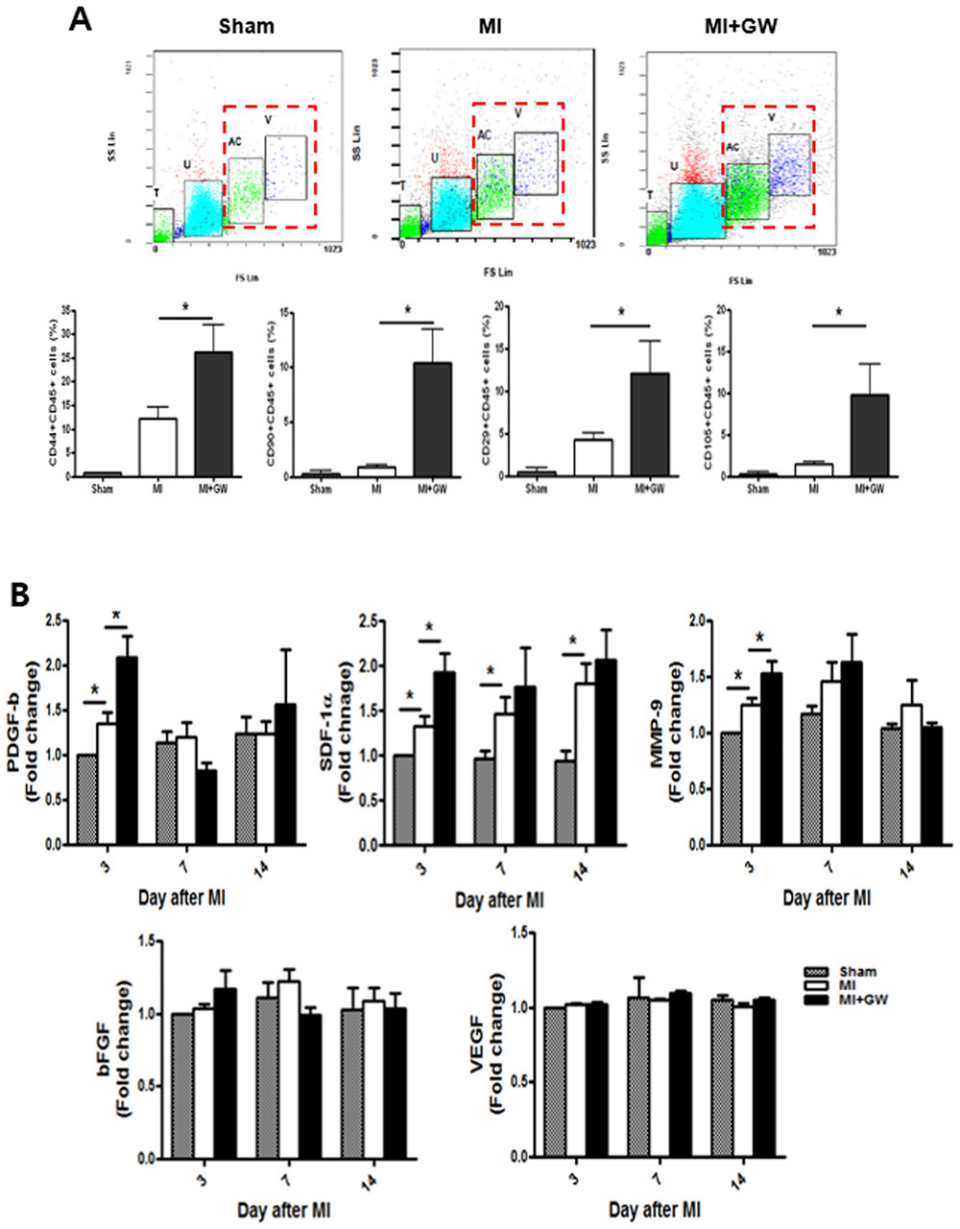
Effects of GW610742 on MSC migration into the infarcted hearts. **(A)** Rat cardiac cells on days 3 after MI were prepared from the ventricles, and adherent cells were stained with each antibody. “T” and “U”-gated cells (floating cells also have “T” and “U”-gated cells) were ruled out for analysis, and “AC” and “V”-gated cells were verified for MSC-phenotype analysis. MSC-positive cells were counted in “AC” and “V”-gated cells. (**B)** Serum from each group on each day was measured for PDGF-b, SDF-1α, MMP-9, bFGF, and VEGF using specific ELISA kit. Values are represented as the mean ± SEM. *P <0.05. MSC, mesenchymal stem cell; MI, myocardial infarction; PDGF-b, platelet-derived growth factor subunit B; MMP-9, matrix metallopeptidase 9; SDF-1α, stromal-derived factor-1 alpha; bFGF, basic fibroblast growth factor; VEGF, vascular endothelial growth factor. Sham (n = 3), MI (n = 7), MI + GW (n = 7).

Recent reports have shown that bone marrow (BM)-derived MSCs or a structural population of MSCs is increased in the heart after MI [21]. We examined whether GW610742 affects the MSC population in the heart after MI. Our results showed that each of the cardiac MSCs that expressed CD44, CD29, CD90, and CD105 were also positive for CD45. This result suggests that after MI, cardiac MSCs might be recruited from the BM. The levels of these cardiac MSCs were much higher on day 3 than they were on day 7 (S6 Fig). Notably, all of the MSC phenotypes on day 3 were significantly increased in the MI + GW group compared to in the MI group (Fig 6A). We also investigated which soluble factor is involved in the migration of BM-MSCs after MI. Compared to the MI group, serum PDGF-b, SDF-1α and MMP-9 levels were significantly increased only on day 3 in the MI + GW group (Fig 6B). The bFGF level was also increased by MI, but this increase was not significant compared to the level in the sham group and was not comparable between the MI and the MI + GW groups. The VEGF level did not change in any of the groups at any of the time points.

### GW610742 increases the differentiation of fibroblasts into myofibroblasts in the infarcted hearts

**Fig 7:**
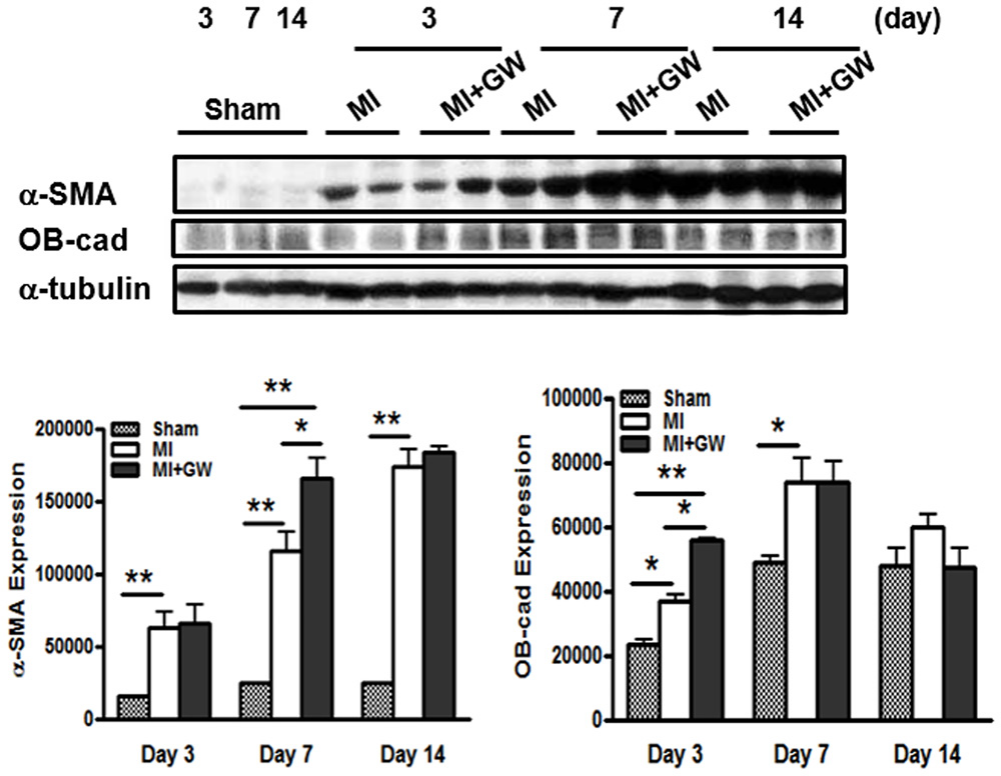
Effects of GW610742 on α-SMA and OB-cadexpression. α-SMA (42 kDa) and OB-cad (120 kDa) expression were assessed by immunoblot analyses in all groups on days 3, 7 and 14 following surgery. Densitometric analysis shows the relative levels of α-SMA and OB-cad expression in each group. α-tubulin (42 kDa), used as a loading control, was not different between the groups. Representative blots are derived from three separate experiments. Values are represented as the mean ± SEM. *P <0.05 and **P < 0.01. OB-cad, osteoblast cadherin; α-SMA, α-smooth muscle actin. Sham (n = 3), MI (n = 10), MI + GW (n = 10).

It is well known that myofibroblasts play an important role in wound healing. A variety of cells, such as fibroblasts and recruited stem cells from remote tissues, differentiate into myofibroblasts, which generate wound tension and architecture [17]. To determine whether GW610742 affects the phenotypic differentiation of fibroblasts into myofibroblasts, we performed immunoblot analysis for α-SMA. The expression level of α-SMA was higher in the MI + GW group than it was in the MI group on day 7 (Fig 7). Particularly, α-SMA expression was well correlated with cardiac fibrosis on day 7 after MI, as shown in Fig 2. OB-cad, a member of the atypical type II cadherin family, is considered specific to the BM-MSC phenotype expression observed in the early healing phase [22]. The expression of OB-cad was significantly increased on day 3 after MI in the MI + GW group compared to the expression in the MI group (Fig 7). The expression levels of α-SMA and OB-cad were not significant at day 14 in both the MI and GW610742-treated MI hearts.

## Discussion

We found that the PPAR-δ agonist GW610742 treatment advances the onset of cardiac fibrosis and angiogenesis in the early infarct site after MI. This PPAR-δ agonist augmented the recruitment of BM-derived MSCs to injured heart tissues, the differentiation of fibroblasts into myofibroblasts. This sequential fibrosis process by PPAR-δ was accompanied by earlier angiogenesis and TGF-β2 overexpression. However, the prolonged pharmacological PPAR-δ activation did not lead to beneficial effects on cardiac function in this study, despite the histological advance of the healing process in the early phase after MI.

During the healing process after MI, the myofibroblasts play critical roles by suppressing the inflammatory reaction and initiating the proliferative phase. The synthesis and deposition of α-SMA and other contractile proteins are important for infarct contraction and structural integrity. Although chronic excessive fibrosis is associated with increased ventricular stiffness and heart failure, prolonged extracellular matrix degradation and a lack of fibrosis in the vulnerable myocardium after MI can lead to LV aneurysms and ruptures, which can worsen LV remodeling [6,7]. Teunissen et al. [9] previously reported findings that are contradictory to our current data, namely that the exposure of rat neonatal cardiac fibroblasts to the PPAR-δ ligand or adenoviral overexpression of PPAR-δ significantly decreased proliferation, collagen synthesis, and the α-SMA level, indicating reduced myofibroblast differentiation *in vitro*. However, treatment with the PPAR-δ agonist after MI *in vivo* might show different effects on wound fibrosis because of different humoral environments, such as elevated TGF-β2, and different origins and characteristics of myofibroblasts from MSCs or adult structural cardiac cells.

Recent studies have shown that PPAR-δ activation inhibits endothelial cells apoptosis and promotes proliferation and angiogenesis [12,23]. Han et al. [12] showed that PPAR-δ activates endothelial progenitor cells (EPCs) through the MMP-9 mediated paracrine network. In the current study, we found that PPAR-δ activation by GW610742 significantly increased angiogenesis reflected by increased CD31 and Ki-67-positive cells on day 7 after MI. However, Ki-67-positive cells were decreased in MI+GW group on day 14. This means that PPAR-δ agonist could advance the angiogenesis under MI circumstances. Furthermore, PPAR-δ expression was naturally elevated in the infarcted heart tissue in the early 3 and 7 day after MI in our study. Therefore, PPAR-δ stimulation might have temporal effects on angiogenesis, myofibroblast differentiation, and fibrosis in *in vivo* MI model. Because the persistence of myofibroblasts in the infarcted heart contributes to progressive cardiac fibrosis, these temporal effects of the PPAR-δ agonist could have a safe and physiological role on infarcted myocardial healing.

We tried to determine the mechanism(s) by which PPAR-δ affords this advanced fibrosis. Fibrosis is initiated by fibroblast proliferation or recruitment in the injured tissue. It has been reported that MSCs contribute to the infarct fibroblast population [21]. We first identified that the MSCs were significantly increased in the infarcted hearts in rats given to GW0742. In a previous study, systemic administration of PPAR-δ agonist enhanced migration of hematopoietic stem cells and EPCs into the bone marrow and peripheral blood, respectively, in mice models [13]. We noted that serum MMP-9, PDGF-b, and SDF-1α, mediators of the recruitment/migration and differentiation MSC migration, also increased in the MI + GW group. These previous reports [12, 24, 25] support our data that PPAR-δ agonist can induce the migration of BM-derived MSCs into the infarcted myocardium after MI. Next, we confirmed the augmented differentiation of fibroblasts into myofibroblasts based on OB-cad and α-SMA expression. Phenotypic differentiation of fibroblasts into myofibroblasts is one mechanism for fibrosis initiation and is a key event in the wound-healing and remodeling processes [26].

Additionally, we observed increased TGF-β2, rather than TGF-β1, expression in the infarcted hearts treated with the PPAR-δ agonist. It is for the first time the marked increase in TGF-β2 expression by the PPAR-δ agonist in the infarcted heart. We also found that TGF-β2 expression was more sensitive to the PPAR-δ agonist than was TGF-β1 expression in skin fibroblasts (data not shown) and activation of PPAR-δ with GW610742 induced α-SMA protein expression in fibroblasts (data not shown). Thus, these results suggest that activation of PPAR-δ might be involved in fibroblast to myofibroblast differentiation. Such an increase in TGF-β2 expression was accompanied by myofibroblast differentiation at repair sites during the healing period (Fig 5 and S2 Fig). No study has examined whether a PPAR response element exists in the promoter region of the *TGF-β2* gene, as it does in the *TGF-β1* gene. In addition, little is known about the biological functions of TGF-β2 during the cardiac healing process.

It is still unclear why histological early fibrosis did not lead to beneficial LV remodeling in our study. PPAR-δ agonist treatment could advance the temporal onset of cardiac fibrosis through MSC recruitment, earlier angiogenesis, and increased differentiation to myofibroblasts, but not enhance the total fibrosis in infarcted myocardium after completion of healing period. These findings suggest that PPAR-δ agonist treatment have only temporal effect on early fibrosis phase after MI, which effect could not sustain during entire healing process. Further study should be performed for defining the therapeutic role and mechanisms of PPAR-δ on infarct healing, naturally overexpressed in early infarcted tissue.

## Supporting Information

S1 Fig The representative relevant images of the changes of morphology after MI. The relevant images represented as infarct area. Infarct areas were calculated based on percentage for LV length with fibrosis/total LV length in same level including the septum (arrow on day 14). Sham, sham-operated; MI, myocardial infarction; MI+GW, MI treated with GW610742. Sham (n = 3), MI (n = 10), MI + GW (n = 10).
(TIF)

S2 Fig Effects of GW610742 on α-SMA expression and cardiac fibrosis. α-SMA (42 kDa) expression was assessed by immunoblot analyses in all groups on day 3, 7, and day 14 following surgery. Densitometric analysis shows the relative levels of α-SMA expression in each group. α-tubulin (42 kDa), used as a loading control, was not different between the groups. Representative blots are derived from three separate experiments. Values are represented as the mean ± SEM. *P < 0.05 and **P < 0.01. Sham (n = 3), MI (n = 10), MI + GW (n = 10) **(Figure A)**. Representative images from sham-operated (Sham), myocardial infarction alone (MI), and MI treated with GW610742 (MI + GW) rats on day 0, 3, 7, and day 14 after MI. Densitometric analysis shows MT staining between the groups **(Figure B)**. Values are represented as the mean ± SEM. *P < 0.05 and **P < 0.01 vs. the corresponding MI group. Scale bars = 100 μm. Sham, sham-operated; MI, myocardial infarction; MI+GW, MI treated with GW610742; MT, Masson’s trichrome stain.
(TIF)

S3 Fig Effects of GW610742 on cardiac angiogenesis on day 0 and day 3 post MI. Representative images from the sham-operated (Sham), myocardial infarction alone (MI), and MI treated with GW610742 (MI + GW) groups on day 0 **(Figure A)** and day 3 **(Figure B)** post-surgery. Images for CD31 and Ki-67 staining show a few vessels in the heart post-operation. The immunoreactivity and positive cell numbers for CD31 and Ki-67 were counted in each group. Values are represented as the mean ± SEM. Scale bars = 100 μm and 50 μm for CD31 and Ki-67 staining, respectively. Sham (n = 3), MI (n = 7), MI + GW (n = 7).
(TIF)

S4 Fig Expression of PPAR-δ in the infarcted zone. PPAR-δ protein expression in the infarct zone in hearts with MI. Representative blots for PPAR-δ expression from sham-operated rats (Sham), rats with MI alone (MI), and MI treated with GW610742 (MI + GW) groups on day 3 and 7 post-surgery. *P < 0.05. PPAR-δ, peroxisome proliferator-activated receptor-delta. Sham (n = 4/each day), MI (n = 10/each day), MI+GW (n = 10/each day).
(TIF)

S5 Fig Effects of GW610742 on TGF-β1 expression. TGF-β1 expression was assessed by immunoblot analyses in all groups on day 3, 7, and day 14 following surgery. Densitometric analysis shows the relative levels of TGF-β1 expression in each group. α-tubulin (42 kDa), used as a loading control, was not different between the groups. Representative blots are derived from three separate experiments. Values are represented as the mean ± SEM. **P < 0.01. Sham (n = 3), MI (n = 10), MI + GW (n = 10).
(TIF)

S6 Fig Effects of GW610742 on MSC migration into the infarcted hearts. Rat cardiac cells on days 3 and 7 after MI were prepared from the ventricles and adherent cells were stained with each antibody. *P < 0.05. Sham (n = 3), MI (n = 7), MI + GW (n = 7).
(TIF)
