# Supplementary figures and images for: Effects of Peroxisome Proliferator-Activated Receptor-δ Agonist on Cardiac Healing after Myocardial Infarction
Source: PLoS One. 2016 Feb 10;11(2):e0148510. doi: 10.1371/journal.pone.0148510 (PMC4749247; doi:10.1371/journal.pone.0148510)

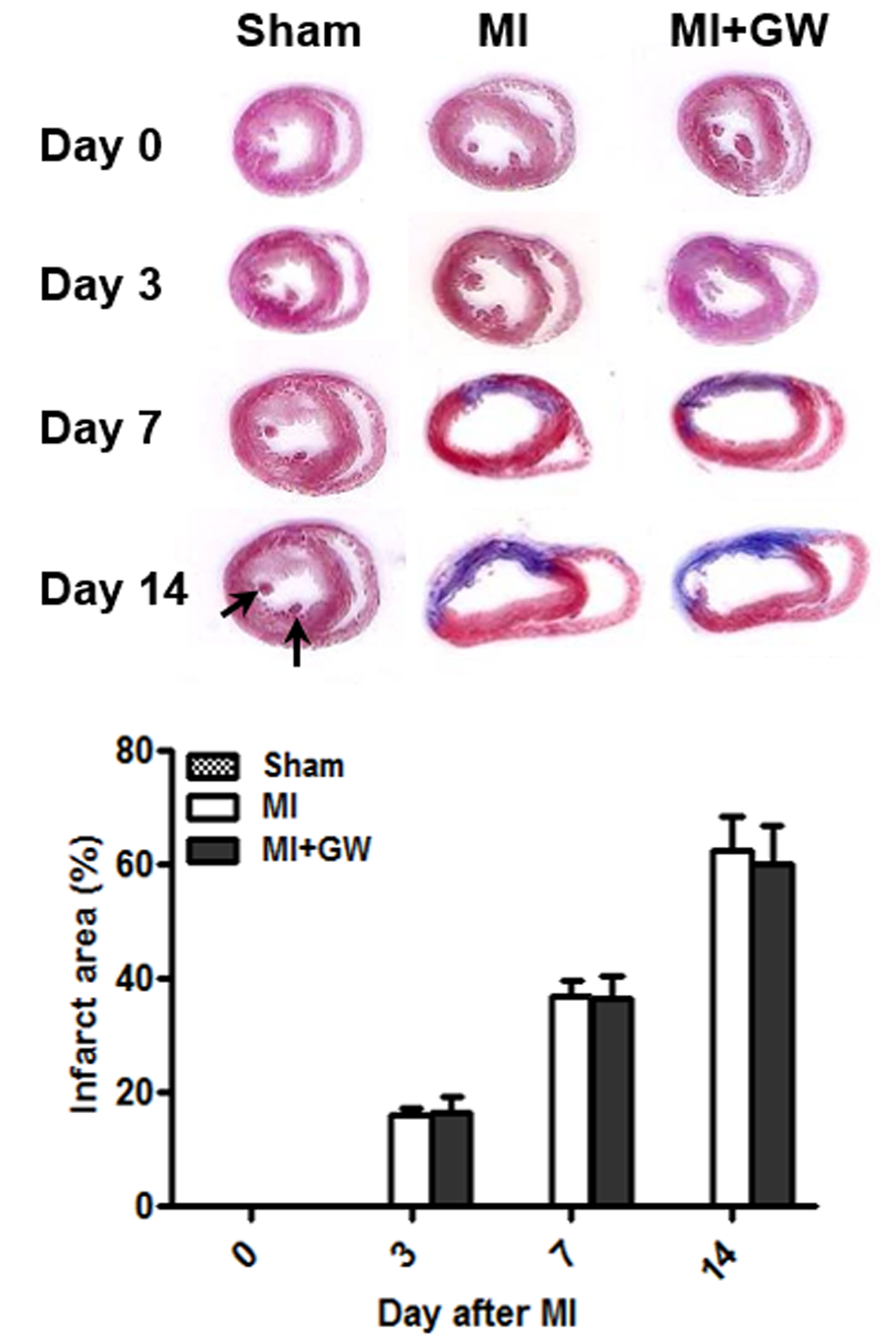

Supplement: S1 Fig — The relevant images represented as infarct area. Infarct areas were calculated based on percentage for LV length with fibrosis/total LV length in same level including the septum (arrow on day 14). Sham, sham-operated; MI, myocardial infarction; MI+GW, MI treated with GW610742. Sham (n = 3), MI (n = 10), MI + GW (n = 10). (TIF) [file pone.0148510.s001.tif]

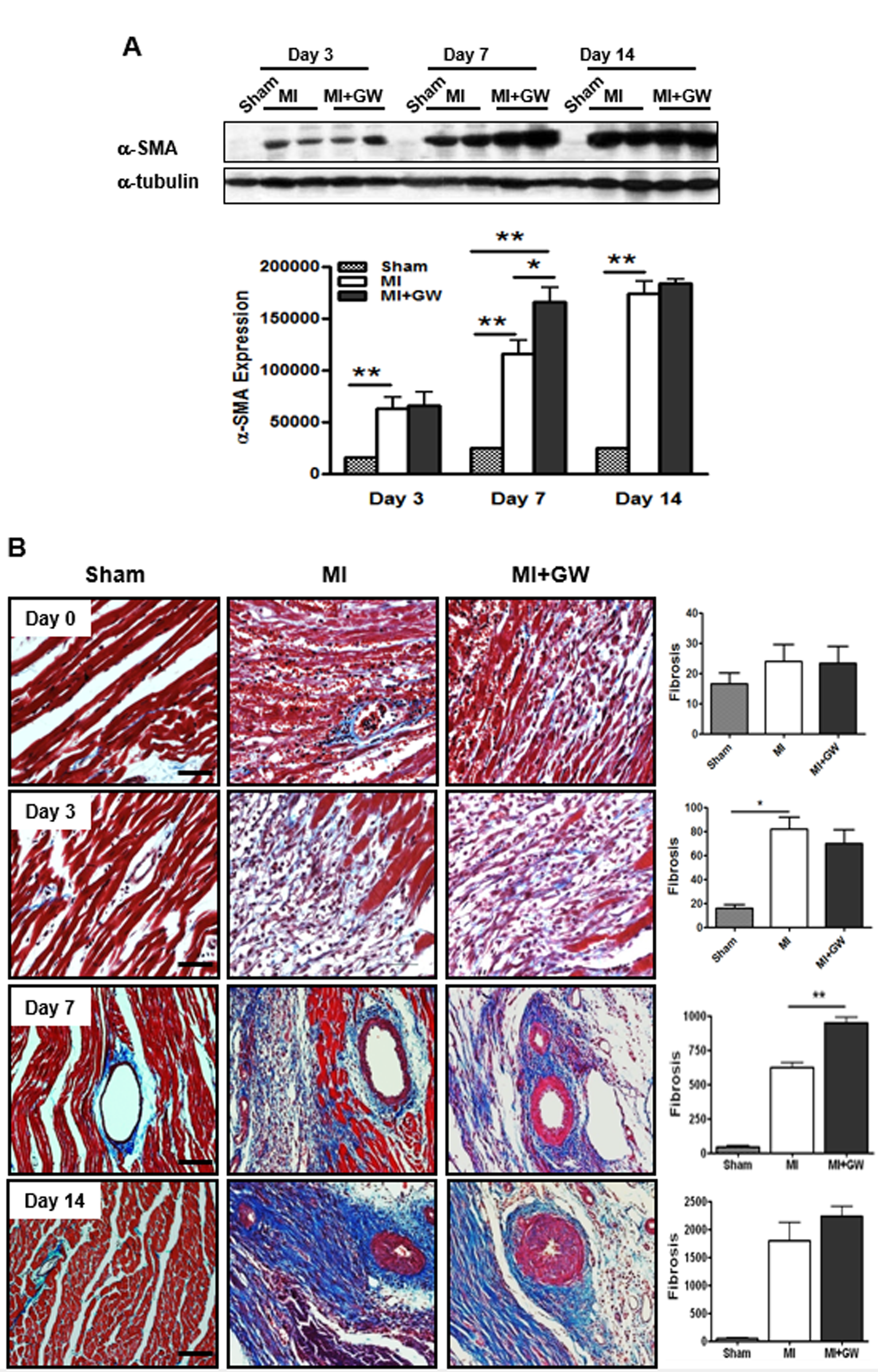

Supplement: S2 Fig — α-SMA (42 kDa) expression was assessed by immunoblot analyses in all groups on day 3, 7, and day 14 following surgery. Densitometric analysis shows the relative levels of α-SMA expression in each group. α-tubulin (42 kDa), used as a loading control, was not different between the groups. Representative blots are derived from three separate experiments. Values are represented as the mean ± SEM. *P < 0.05 and **P < 0.01. Sham (n = 3), MI (n = 10), MI + GW (n = 10) (Figure A). Representative images from sham-operated (Sham), myocardial infarction alone (MI), and MI treated with GW610742 (MI + GW) rats on day 0, 3, 7, and day 14 after MI. Densitometric analysis shows MT staining between the groups (Figure B). Values are represented as the mean ± SEM. *P < 0.05 and **P < 0.01 vs. the corresponding MI group. Scale bars = 100 μm. Sham, sham-operated; MI, myocardial infarction; MI+GW, MI treated with GW610742; MT, Masson’s trichrome stain. (TIF) [file pone.0148510.s002.tif]

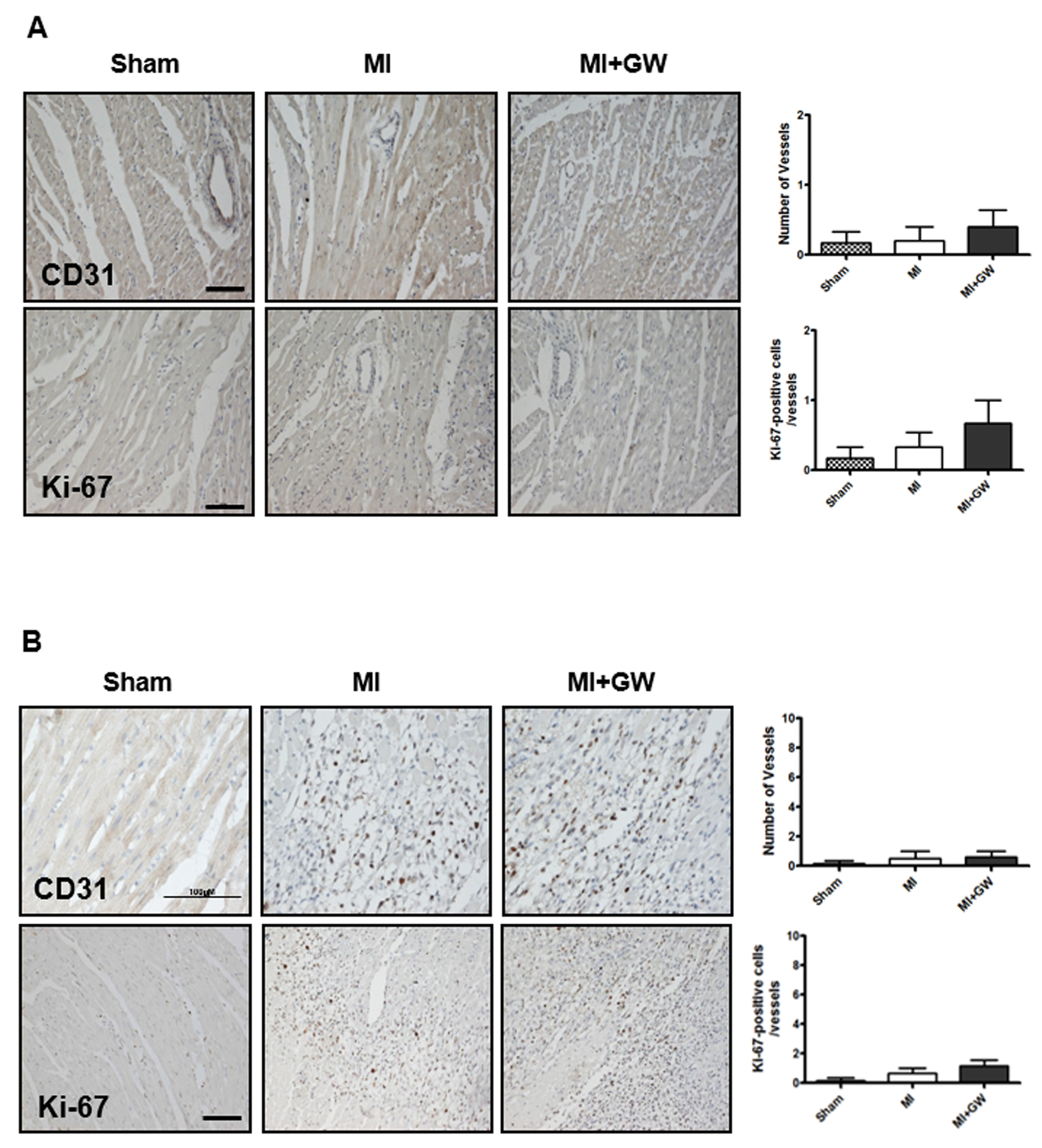

Supplement: S3 Fig — Representative images from the sham-operated (Sham), myocardial infarction alone (MI), and MI treated with GW610742 (MI + GW) groups on day 0 (Figure A) and day 3 (Figure B) post-surgery. Images for CD31 and Ki-67 staining show a few vessels in the heart post-operation. The immunoreactivity and positive cell numbers for CD31 and Ki-67 were counted in each group. Values are represented as the mean ± SEM. Scale bars = 100 μm and 50 μm for CD31 and Ki-67 staining, respectively. Sham (n = 3), MI (n = 7), MI + GW (n = 7). (TIF) [file pone.0148510.s003.tif]

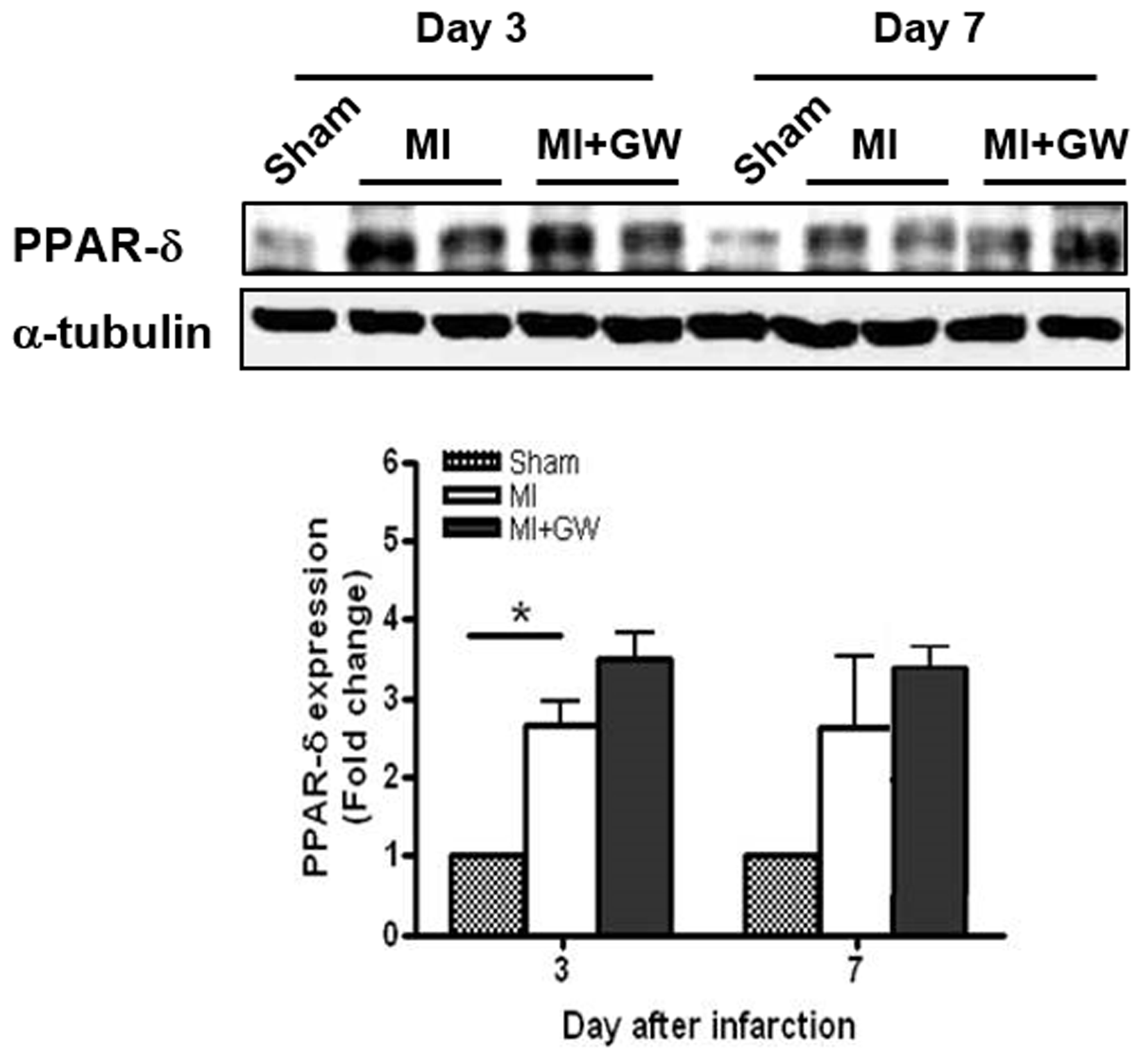

Supplement: S4 Fig — PPAR-δ protein expression in the infarct zone in hearts with MI. Representative blots for PPAR-δ expression from sham-operated rats (Sham), rats with MI alone (MI), and MI treated with GW610742 (MI + GW) groups on day 3 and 7 post-surgery. *P < 0.05. PPAR-δ, peroxisome proliferator-activated receptor-delta. Sham (n = 4/each day), MI (n = 10/each day), MI+GW (n = 10/each day). (TIF) [file pone.0148510.s004.tif]

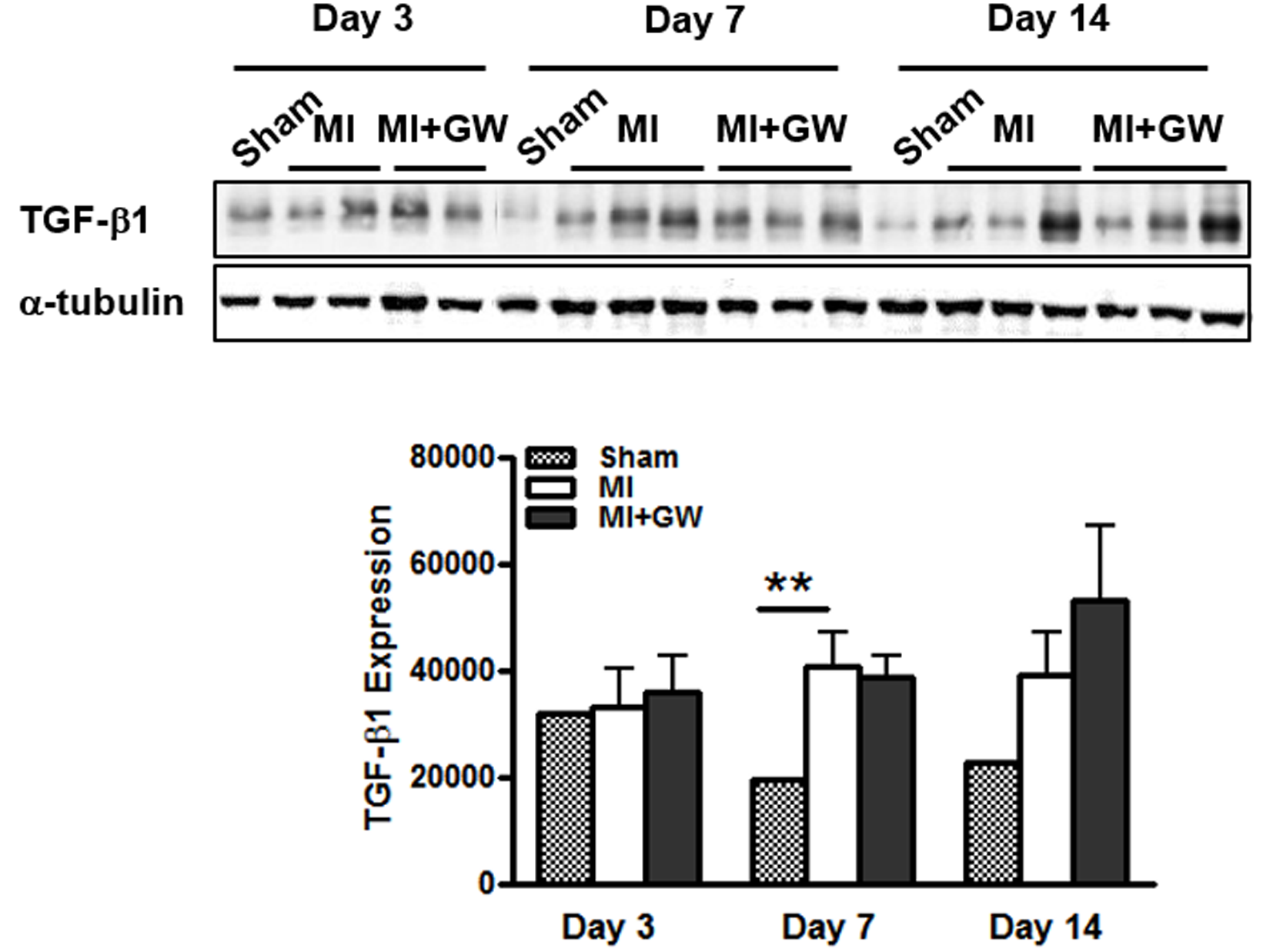

Supplement: S5 Fig — TGF-β1 expression was assessed by immunoblot analyses in all groups on day 3, 7, and day 14 following surgery. Densitometric analysis shows the relative levels of TGF-β1 expression in each group. α-tubulin (42 kDa), used as a loading control, was not different between the groups. Representative blots are derived from three separate experiments. Values are represented as the mean ± SEM. **P < 0.01. Sham (n = 3), MI (n = 10), MI + GW (n = 10). (TIF) [file pone.0148510.s005.tif]

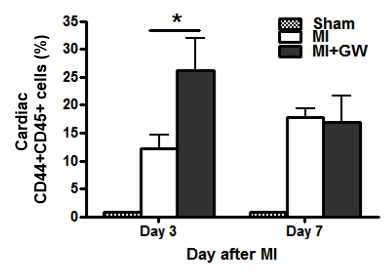

Supplement: S6 Fig — Rat cardiac cells on days 3 and 7 after MI were prepared from the ventricles and adherent cells were stained with each antibody. *P < 0.05. Sham (n = 3), MI (n = 7), MI + GW (n = 7). (TIF) [file pone.0148510.s006.tif]
